# Identification of a key ceRNA network associated with ferroptosis in gastric cancer

**DOI:** 10.1038/s41598-022-24402-3

**Published:** 2022-11-22

**Authors:** Wen Jin, Jianli Liu, Jie Yang, Zongqi Feng, Zhenxing Feng, Na Huang, Tingyu Yang, Lan Yu

**Affiliations:** 1 Clinical Medical Research Center/Inner Mongolia Key Laboratory of Gene Regulation of the Metabolic Disease, Inner Mongolia People’s Hospital, Hohhot, 010010 China; 2 School of Water Resource and Environment Engineering, China University of Geosciences, Beijing, 100083 China; 3 College of Sciences, Inner Mongolia University of Technology, Hohhot, 010051 China

**Keywords:** Data mining, Data processing, Gene regulatory networks, Genome informatics, Predictive medicine

## Abstract

Ferroptosis, a newly discovered irondependent form of regulated cell death caused by excessive accumulation of lipid peroxides, is linked to the development and treatment response of various types of cancer, including gastric cancer (GC). Noncoding RNAs (ncRNAs), as key regulators in cancer, have both oncogenic and tumor suppressive roles. However, studies on ferroptosis-related ncRNA networks in GC are still lacking. Here, we first identified 61 differentially expressed genes associated with ferroptosis in GC by computing and analyzing gene expression profile of tumor and normal tissues for GC. Then, upstream lncRNAs and miRNAs interacting with them were found through miRNet and miRBase databases, and hub lncRNAs and miRNAs were obtained through topological analysis. Finally, the ceRNA regulatory network linked to ferroptosis in GC was established, which includes two ferroptosis marker genes (*TXNIP* and *TSC22D3*), one driver gene (*GABARAPL1*), and one suppressor gene (*CAV1*). Kaplan-Meier survival analysis showed that changes in the expression of these genes were associated with the survival of GC patients. Furthermore, our study revealed that this ceRNA network may influence the progression of GC by regulating ferroptosis process. These results will help experimental researchers to design an experiment study to further explore the roles of this regulatory network in GC ferroptosis.

## Introduction

Ferroptosis, a newly discovered mechanism and unique modality of programmed cell death, is driven by the accumulation of a large number of iron, free radical production, fatty acid supply, lipid peroxidation, and the destruction of intracellular redox balance^1,2^. It is an irondependent manner of nonapoptotic cell death. Many cellular biological processes can alter cellular susceptibility to ferroptosis by altering cellular iron contents^3^. For example, enhancing intracellular iron export makes some cells more resistant to ferroptosis^4,5^. A study suggests that when transferrin expression is silenced, hepatocytes compensatorily upregulate *SLC39A14* expression, resulting in excessive import of iron that then leads to ferroptosis^6^. Additionally, numerous signaling pathways and cellular metabolic pathways relevant to human disease, notably cancer, are driven by ferroptosis^7,8^. A growing number of studies have confirmed that ferroptosis has been involved in the development of a variety of cancer, and plays a critical role in inhibiting tumorigenesis^2,9^. However, research into ferroptosis have just begun in many ways, and the underlying mechanism of ferroptosis in cancers, particularly gastric cancer, is poorly understood.

Gastric cancer (CG) is a common and deadly human malignancy that seriously threatens the lives and health of millions of people worldwide^10,11^. With the rapid development of medical technology, the diagnosis and treatment of GC have made great progress, but patient survival is still poor^12,13^. Precision therapy remains a challenge. More recently, accumulating evidence indicates that ferroptosis is critical for eliminating cancer cells, which could open up a new way for cancer therapy^9,14^.

Interestingly, long noncoding RNA (lncRNA) and microRNA (miRNA) are increasingly recognized as crucial regulators in the regulation of ferroptosis of cancer cells^9,15^. The regulatory loop between lncRNA and miRNA plays a dynamic role in transcription and translation of protein-coding genes, influencing multiple biological processes in cancers, such as cell death, cell cycle and proliferation^16–18^. lncRNAs function as competitive endogenous RNAs (ceRNAs) to regulate mRNAs through competitively binding miRNAs, therefore forming largescale regulatory networks across the transcriptome^17^. CeRNA regulatory networks play important roles in cancer initiation and development^19–21^, as well as have significant regulatory effects on the ferroptosis of cancer^22^. lncRNA *NEAT1*, as a ceRNA, facilitates ferroptosis in hepatocellular carcinoma by controlling miR-362-3p and *MIOX*^23^. MiR-375 can trigger ferroptosis to suppress the stemness of GC cells through interacting *SLC7A11*, which could be used as a potential target to induce ferroptosis^24^. However, the function and molecular mechanism of ncRNAs in regulating ferroptosis of cancers remain unclear.

To understand the relationship between ncRNAs and ferroptosis in gastric cancer, we constructed a ceRNA network related to ferroptosis, which revealed the underlying mechanism of lncRNAs and miRNAs in regulating ferroptosis of GC. First, aberrantly expressed genes associated with ferroptosis were obtained in GC by transcriptome analysis. Further, functional and pathway enrichment analyses were conducted to explore the roles of these genes in GC. Then, upstream lncRNAs and miRNAs that affected the abnormal expression of ferroptosis-related genes were identified, and the four key lncRNAs and six miRNAs were found by topological analysis. Finally, these four key lncRNAs were revealed to act as ceRNAs to modulate ferroptosis in gastric cancer by regulating cancer-related miRNAs and protein-coding genes. Collectively, our findings provide new insights into the regulation of ferroptosis as a mean of eliminating gastric cancer cells.

## Results

### Identification of ferroptosis-related genes in gastric cancer

**Figure 1:**
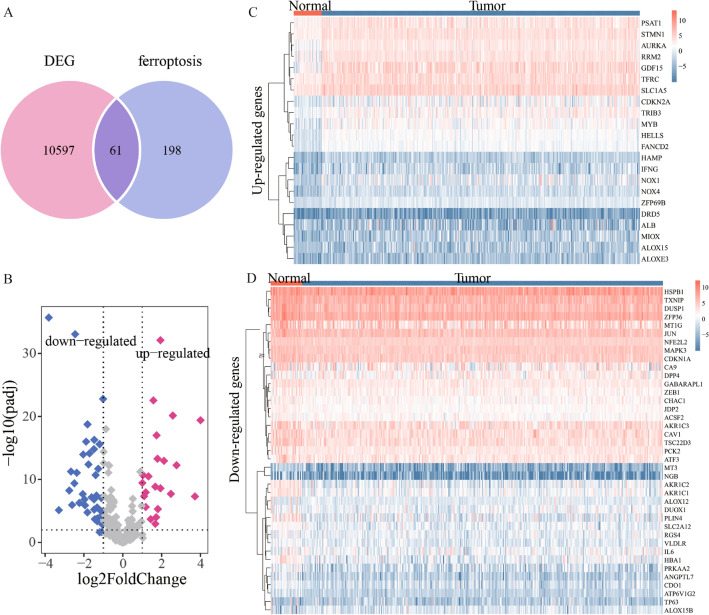
(**A**) The differentially expressed genes (DEGs) associated with ferroptosis in gastric cancer. (**B**) The upregulated genes with padj < 0.05 and log2FoldChang > 1 and the downregulated genes with padj < 0.05 and log2FoldChang < (−1). (**C** and **D**) Heatmaps of 22 upregulated genes and 39 downregulated genes in 343 tumor and 30 normal tissues from GC. The row represents the gene expression value of each gene, and the column represents each sample. The redder the color, the higher the gene expression value. The bluer the color, the lower the gene expression value.

Based on transcriptome data of gastric cancer from the TCGA Cohort, we analyzed significantly differentially expressed genes between the 343 tumor and 30 normal tissues using the negative binomial distribution. The 10,658 unique genes were identified. Among them, the 61 ferroptosis-related genes are dysregulated in GC (Fig. 1A and Table.SI in Supplementary Information 2). 22 of these 61 genes are upregulated (padj < 0.05 and log2FoldChang > 1) and 39 genes are downregulated in gastric cancer (padj < 0.05 and log2FoldChang < (−1); Fig. 1B). Their expression in 343 tumor and 30 normal tissue samples is shown in Fig. 1C,D.

**Figure 2:**
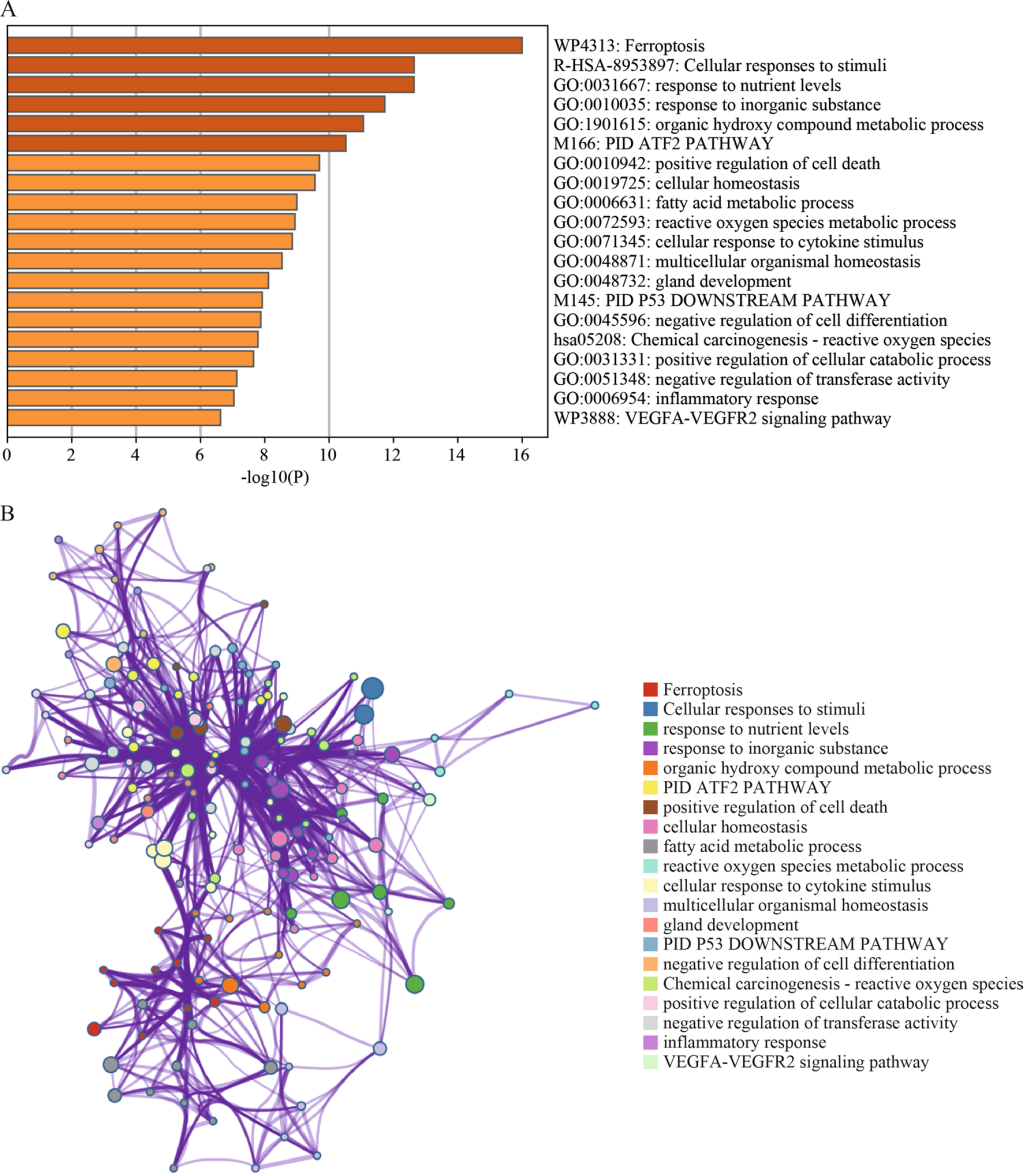
The functional and pathway enrichment analysis of 61 differentially expressed ferroptosis-related genes. (**A**) The top 20 enriched ontology clusters. The abscissa represents the significant *P* value of enrichment. The color represents the *P* value, and darker colors indicate smaller *P* values. (**B**) Network of functional and pathway enrichment terms. Each node represents an enriched term and is colored by its cluster ID. Those nodes that share the same cluster ID are generally close to each other.

In order to further understand the biological behavior of these 61 differentially expressed ferroptosis-related genes in GC, we performed the pathway and biological process enrichment analysis, such as KEGG Pathway, WikiPathways, GO Biological Processes, Reactome Gene Sets, and Canonical Pathways, by Metascape^25^ (Fig. 2 and Table.S1). The result showed that ferroptosis pathway is the most representative enriched term, with Log10 (*p*) equals (−16.00) and Log10 (q) equals (−11.65) (Fig. 2A), which including *CAV1*, *TXNIP*, *DPP4*, *SLC1A5*, *TFRC*, *NOX1*, and *NOX4* (Table.S1). It has been indicated that *NOX4* elevation can promote ferroptosis of astrocyte by activating oxidative stressinduced lipid peroxidation and impairing mitochondrial metabolism in Alzheimer's disease^26^ In addition, ferroptosis pathway was revealed to be closely related to multiple biological processes, such as positive regulation of cell death, fatty acid metabolic process, and reactive oxygen species metabolic process (Fig. 2B). Moreover, Chemical carcinogenesisreactive oxygen species and VEGFA-VEGFR2 signaling pathway were enriched. These data suggested that the abnormal expression of these 61 genes is associated with ferroptosis in gastric cancer, and is involved in other biological processes and signaling pathways related to tumor.

### Identification of upstream lncRNAs and miRNAs associated with ferroptosis

**Figure 3:**
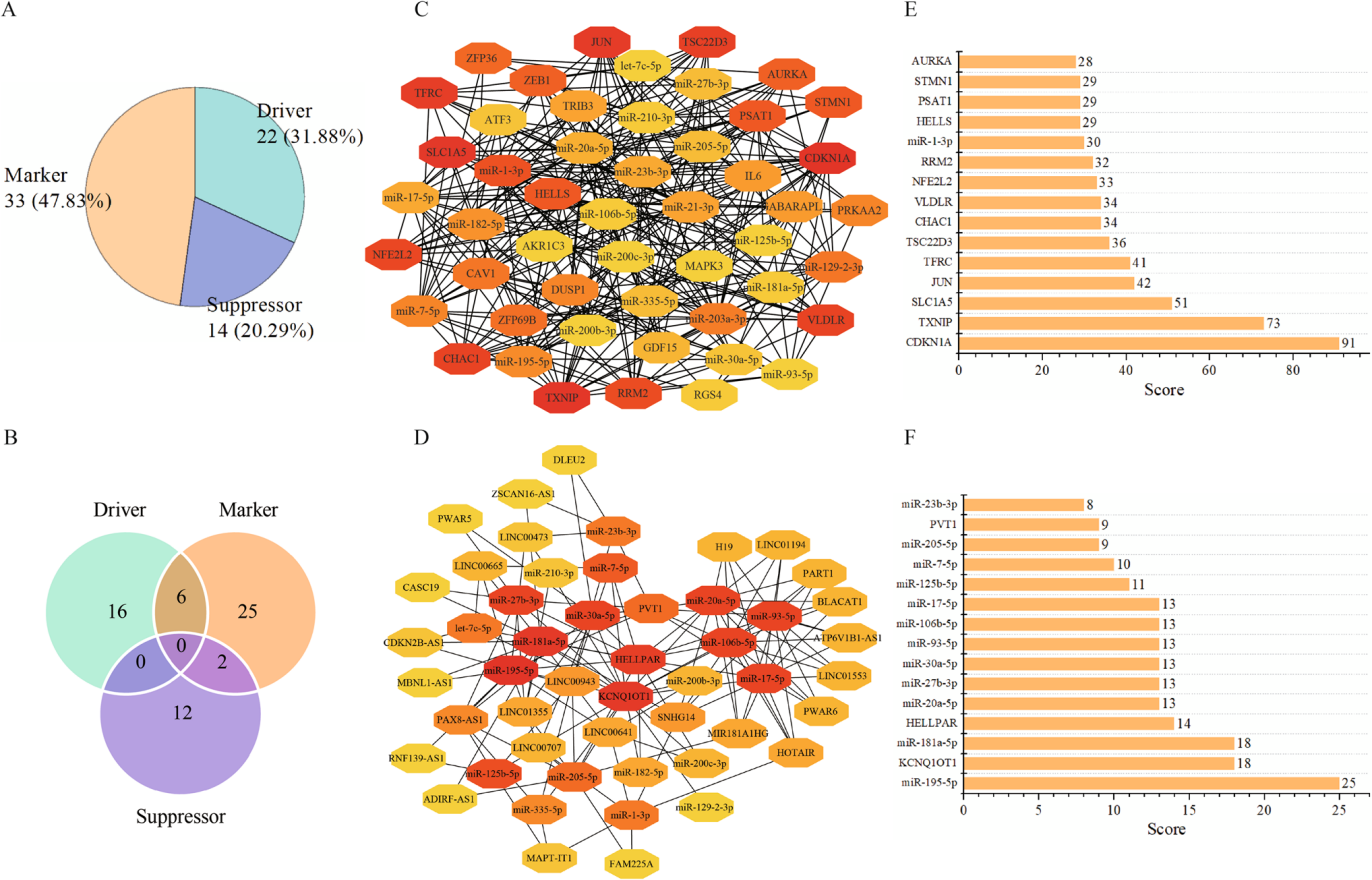
(**A**) Classification of differentially expressed genes associated with ferroptosis in gastric cancer. (**B**) Intersection of driver genes, suppressor genes, and marker genes. (**C**) The hub subnetwork between the 242 DEmiRNAs and 57 ferroptosis-related genes. (**D**) The hub subnetwork between the 20 DEmiRNAs and 83 DElncRNAs. The color of the point represents the connectivity of the node in the network. The darker the color of the nodes, the more important the genes in the network. (**E**) The top 15 key DEmiRNAs/ferroptosis-related genes in the interaction network between the 242 DEmiRNAs and 57 ferroptosis-related genes. (**F**) The top 15 key DEmiRNAs/DElncRNAs in the interaction network between the 20 DEmiRNAs and 83 DElncRNAs.

According to the information provided by the FerrDb database, 22 of the 61 ferroptosis-related genes were identified as driver genes that can promote ferroptosis, such as *MAPK3* and *GABARAPL1*; 14 genes were considered as suppressors, which can prevent ferroptosis, such as *CDKN1A* and *CAV1*; 33 genes were known as markers that can indicate the occurrence of ferroptosis, such as *NFE2L2* and *TXNIP* (Fig. 3A). Among them, some genes play multiple roles in ferroptosis, with 6 genes in both drivers and markers, and 2 genes in both suppressors and markers (Fig. 3B).

In order to understand the role of ncRNAs in the ferroptosis of gastric cancer, we predicted upstream lncRNAs and miRNAs that interact with 61 ferroptosis-related genes. Based on the miRNet and miRBase databases, thousands of upstream miRNAs were found. Among them, 242 miRNA are significantly differentially expressed between tumor and normal tissues from GC (*p* < 0.01 and |log2FoldChang|> 1; Table.SII in Supplementary Information 3).These 242 miRNAs can bind to 57 ferroptosis-related genes, forming 992 interaction pairs. A hub subnetwork consisting of 22 differentially expressed miRNAs (DEmiRNAs) and 28 ferroptosis-related genes was constrcuted using the topological methods in CytoHubba (Fig. 3C). These DEmiRNAs and ferroptosis-related genes have a relatively high degree and play a key role in the network. The top 15 key DEmiRNAs and genes with higher degrees were listed, such as *CDKN1A*, *TXNIP*, and miR13p (Fig. 3E).

Additionally, the upstream lncRNAs associated with ferroptosis were predicted through the miRNet database. 433 lncRNAs were found to interact with 20 of the 22 DEmiRNAs. Among these lncRNAs, 83 lncRNAs are significantly differentially expressed between tumor and normal tissue from GC patients (padj < 0.05 and |log2FoldChang|> 1; Table.SIII in Supplementary Information 4). Based on the interaction between the 20 DEmiRNAs and 83 differentially expressed lncRNAs (DElncRNAs), a hub lncRNAmiRNA subnetwork was constructed by topological method. The network consists of 30 DElncRNAs and 20 DEmiRNAs (Fig. 3D). The top 15 key DElncRNAs and DEmiRNAs were displayed as in Fig. 3F. These key DElncRNAs and DEmiRNAs may play important roles in ferroptosis of gastric cancer.

### Association between ferroptosis-related genes and survival of gastric cancer

**Figure 4:**
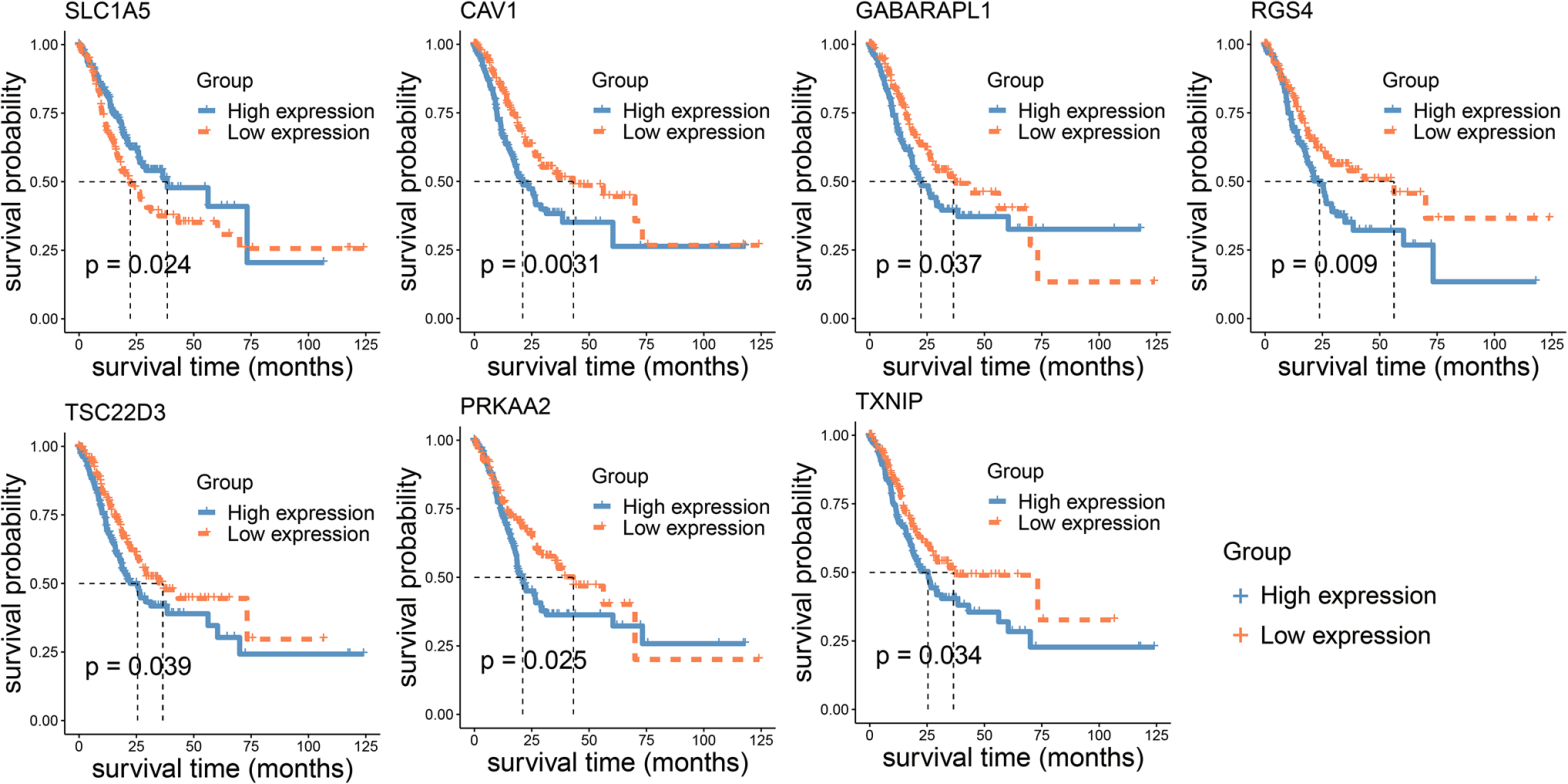
The Kaplan-Meier survival analysis of ferroptosis-related genes. The *p* value was calculated by the log-rank test.

To explore whether these ferroptosis-related genes affect the survival of GC patients, we performed Kaplan-Meier survival analysis. For the 28 key ferroptosis-related genes in hub network, seven genes were found to be associated with patient survival (*p* < 0.05; Fig. 4). The results showed that the five-year survival rate of *SLC1A5* high expression group is higher than that of the low expression group, whereas the five-year survival rate of high expression group of other six genes is lower than that of the low expression group (Fig. 4). For example, patients with high expression of *RGS4* and *TXNIP* exhibited a poorer prognosis in gastric cancer, while those with high expression of *SLC1A5* have a better prognosis within five years.

### Construction of ferroptosis-related ceRNA network in gastric cancer

**Figure 5:**
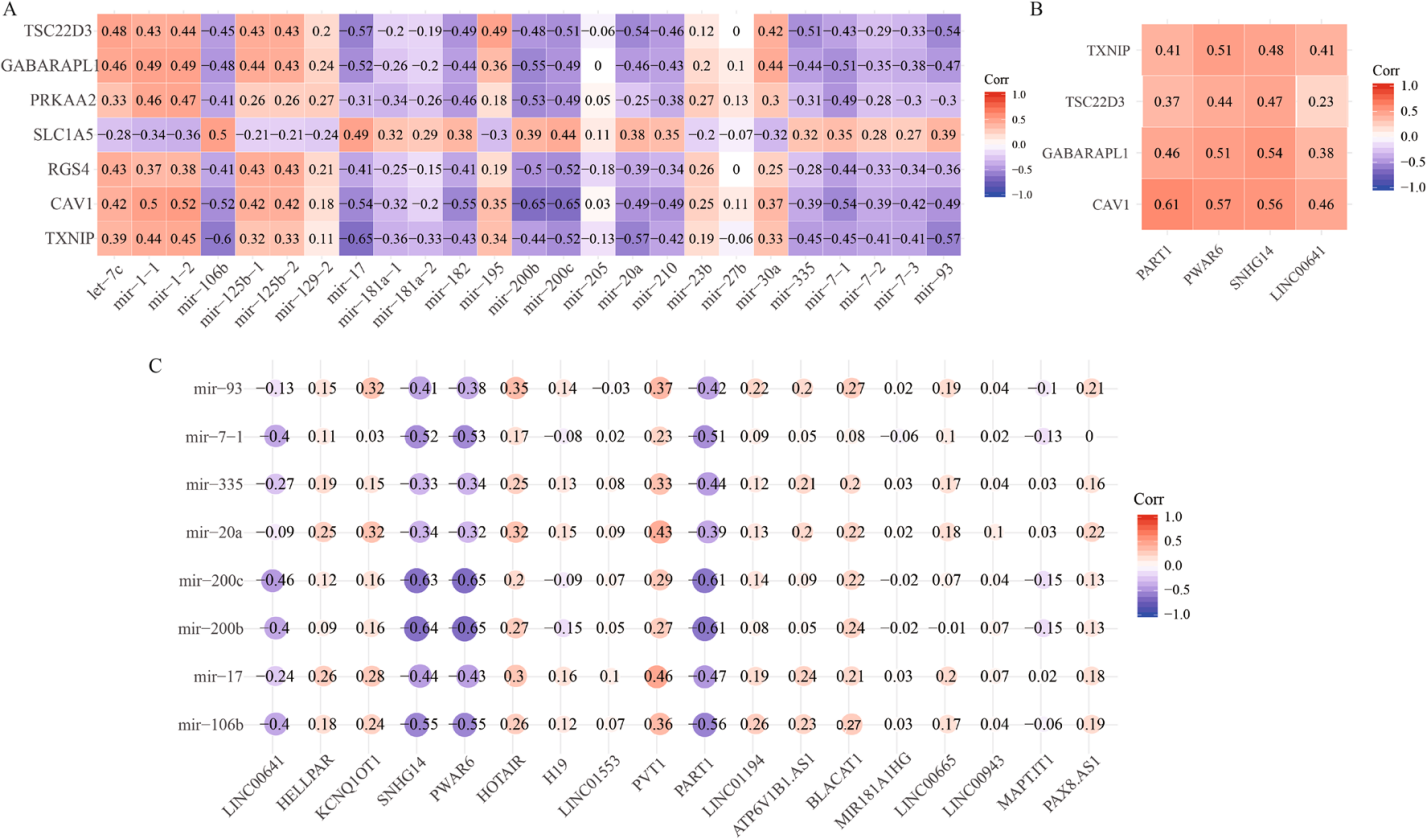
Correlation between the expression of ncRNAs and ferroptosis-related genes in gastric cancer. (**A**) Pearson correlation between the expression level (log2-transformed RPM/FPKM value) of upstream DEmiRNAs and survival-related genes. (**B**) Pearson correlation between the expression level (log2-transformed FPKM value) of upstream DElncRNAs and ferroptosis-related genes. (**C**) Pearson correlation between the expression level (log2-transformed FPKM/RPM value) of upstream DElncRNAs and DEmiRNAs associated with ferroptosis. Corr: Pearson correlation coefficient.

Based on the ceRNA hypothesis, lncRNAs/mRNAs, as ceRNA molecules, function through miRNA response element (MRE) to competitively bind with same miRNAs, thereby regulating gene expression to affect cell function^17,27^. We analyzed the correlation between the expression of seven survival-related genes and DEmiRNAs from the hub subnetwork (Fig. 3C), through the Pearson correlation coefficient. The miRNA-mRNA interaction pairs with Corr < 0.5 and *p* < 0.05 were selected, which include the 8 miRNAs and 4 ferroptosis-related genes (Fig. 5A and Table S2).

Furthermore, Pearson correlation coefficient between the expression of these 8 miRNAs and DElncRNAs from the hub subnetwork (Fig. 3D) was calculated. lncRNA *LINC00641*, *SNHG14*, *PWAR6*, and *PART1* showed negative correlation with the 8 miRNAs, six of which were statistically significant correlated to these lncRNAs (*p* < 0.05; Fig. 5C and Table S3). Moreover, these four lncRNAs interacted with the six miRNAs. In addition, these four lncRNAs were significantly positively correlated with the four ferroptosis-related genes (*p* < 0.05; Fig. 5B).

**Figure 6:**
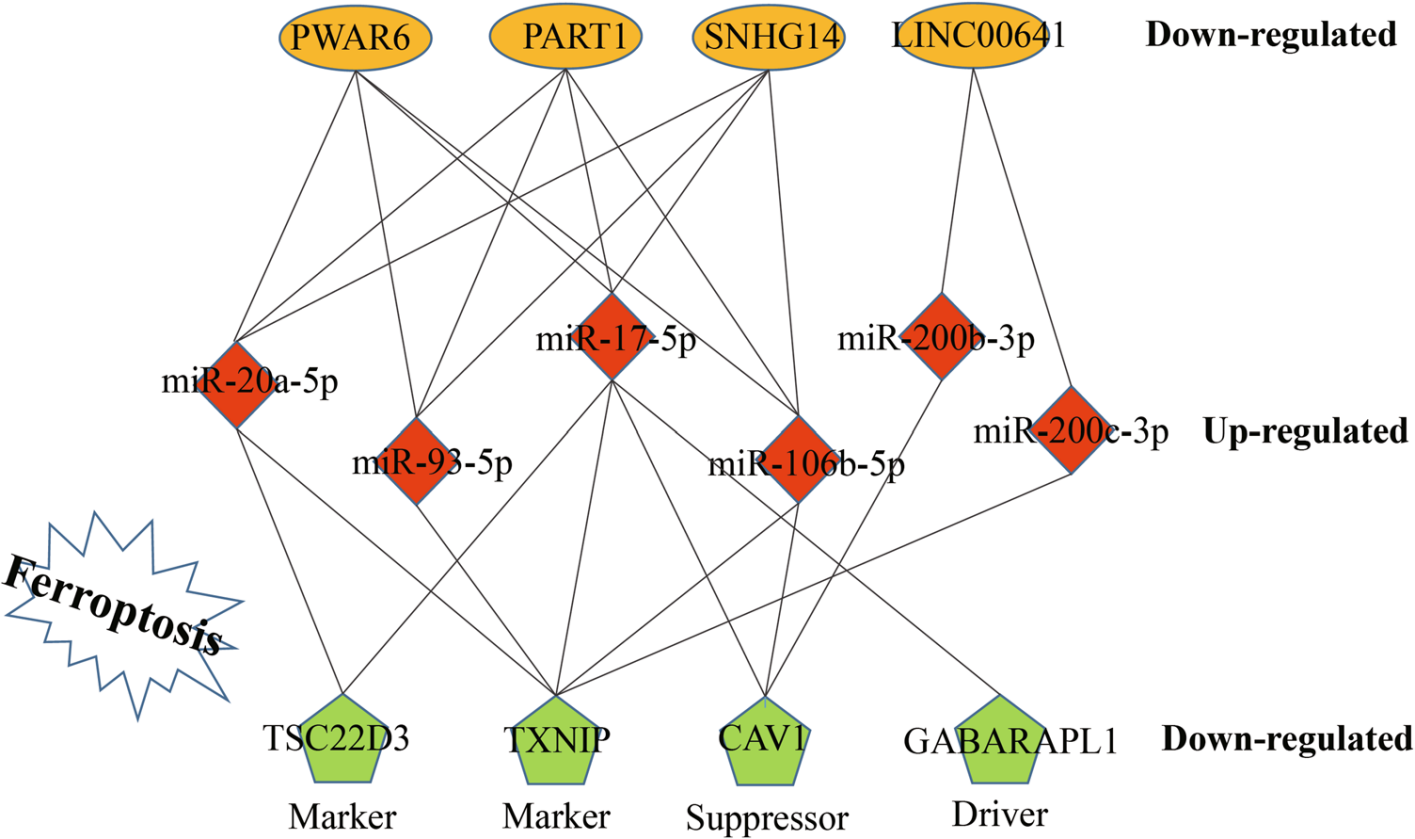
A ferroptosis-related ceRNA regulatory network in gastric cancer.

According to the above analysis, a ferroptosis-related ceRNA regulatory network was constructed by Cytoscape (Fig. 6), which contributes to understand the regulatory role of ncRNAs in ferroptosis of gastric cancer. In this network, the six key DEmiRNAs are shared by four key DElncRNAs (*LINC00641*, *SNHG14*, *PWAR6*, and *PART1*) and ferroptosis-related genes (*CAV1*, *TXNIP*, *GABARAPL1*, and *TSC22D3*). Moreover, the expression of these four DElncRNAs and four ferroptosis-related genes is significantly lower in GC than in normal tissues (Fig.S1). Conversely, the expression of six DEmiRNAs is markedly downregulated in GC tissues (Fig.S2). These data suggested that the four key ferroptosis-related genes are regulated by four key lncRNAs and six key miRNAs, thereby affecting ferroptosis in gastric cancer.

### Validation of a potential ceRNA regulatory network associated with ferroptosis in gastric cancer

**Figure7:**
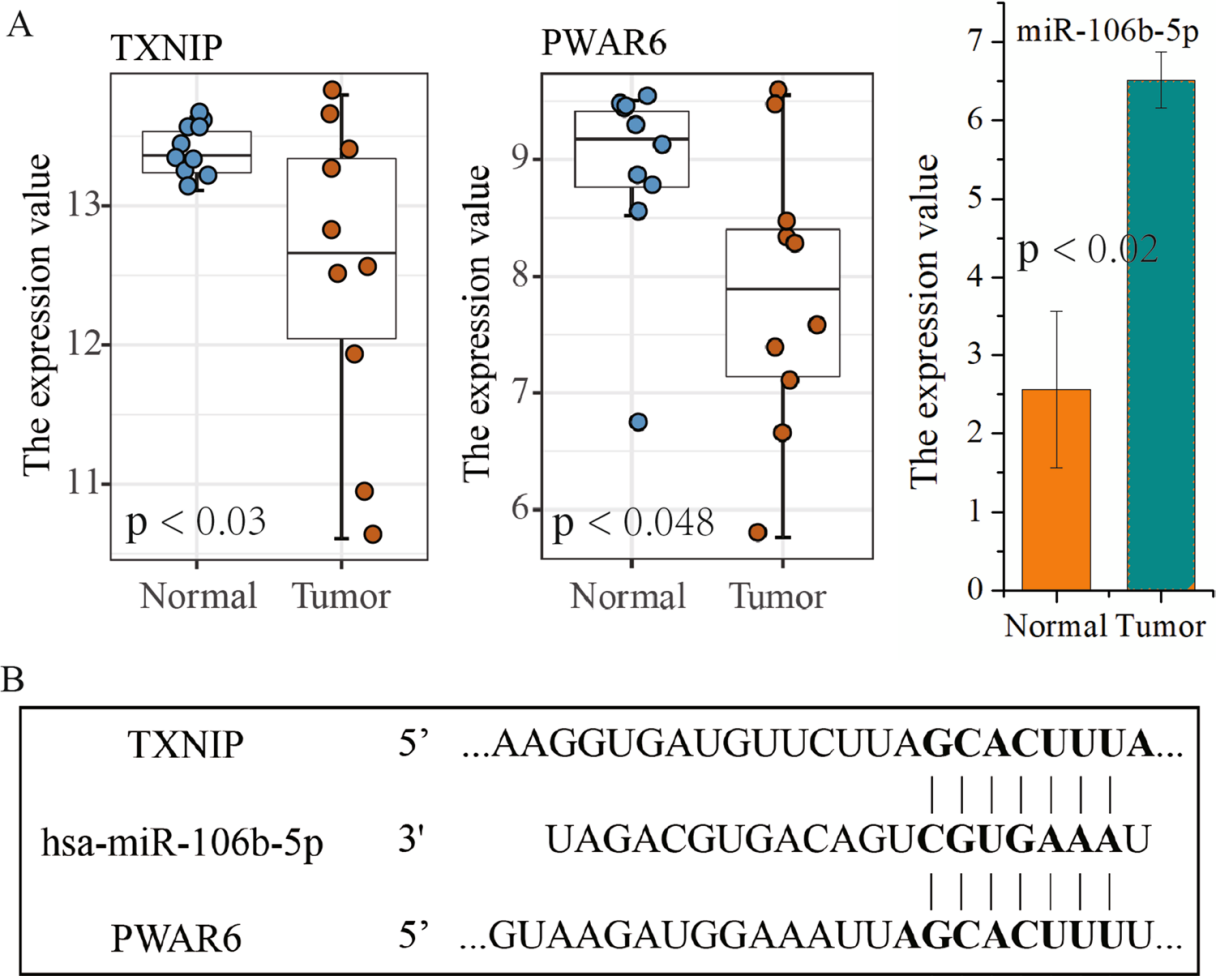
The validation of a ferroptosis-related ceRNA network. (**A**) The expression of *TXNIP* and lncRNA *PWAR6* is downregulated in GC tissues compared to normal tissues, and miR-106b-5p is upregulated in GC. The *p* value was calculated using t-test. (**B**) The binding sites of shared miR-106b-5p with *TXNIP* and *PWAR6*.

In the ferroptosis-related ceRNA regulatory network (Fig. 6), we found that *TXNIP*, a marker of the occurrence of ferroptosis, can be regulated through the interaction of lncRNA *PWAR6* and miR-106b-5p. To further assess the reliability of the results, we analyzed the expression of *TXNIP* and lncRNA *PWAR6* using GSE79973 GC dataset from the GEO database, as well as the expression of shared miR-106b-5p using GSE78091 GC dataset. These data indicated that the expression of *TXNIP* and *PWAR6* is significantly reduced in GC tissues compared to normal tissues, and the expression of miR-106b-5p is markedly elevated in GC (*p* < 0.05; Fig. 7A). The target sites in *TXNIP* and *PWAR6* were predicted to pair with miR-106b-5p by TargetScan analysis (*p* < 0.05; Fig. 7B). These results further demonstrate that lncRNA *PWAR6*, as a competitive endogenous RNA, affects the expression of *TXNIP* by competitively binding miR-106b-5p, which plays a critical role in the ferroptosis of GC.

## Discussion

Ferroptosis is a recently emerged irondependent nonapoptotic cell death cascade that can eliminate cancer cells in a nonapoptotic manner, and is considered a key target for the development of anticancer therapies^7,28^. However, the mechanism that regulates ferroptosis remains unclear. Therefore, the discovery of key factors regulating ferroptosis in cancer has great clinical implications.

With recent advances in research into cancer biology, accumulating studies have shown that ncRNAs, especially lncRNAs and miRNAs, are important mediators in the regulation of ferroptosis and iron metabolism^29,30^. In this study, we obtained 22 upregulated and 39 downregulated genes associated with ferroptosis in gastric cancer, and identified the upstream DElncRNAs and DEmiRNAs that interact with these genes. For example, *SLC1A5* was found to be an upregulated gene associated with ferroptosis in GC and related to patient prognosis (Fig. 1 and 4), which is consistent with the study of *Xiang *et al.^31^. Moreover, both our work and the study by *Xiang *et al. showed that hsa-miR-125b-5p can target *SLC1A5* (Fig. 3), which may play an important role in the targeted therapy of GC. Besides *SLC1A5,* our study also found that 6 of the 61 abnormally expressed ferroptosis-related genes were associated with the prognosis of GC, which include *CAV1*, *GABARAPL1*, *TSC22D3*, *PRKAA2*, *RGS4*, and *TXNIP* (Fig. 4). The study further revealed that *TXNIP*, *CAV1*, *GABARAPL1*, and *TSC22D3* may play key roles in the regulation of ferroptosis in GC. *TXNIP*, a metabolic protein, has been considered to be a tumor suppressor gene in various malignant tumors, and its overexpression can suppress the growth and metastasis of cancer cells in tumor transplant models^32^. *TXNIP* is downregulated in GC than in normal tissues and has been shown to be a key marker for the prognosis of patients with gastric cancer^33^. We confirmed similar results by multiple statistical methods and KM analysis. However, to our knowledge, few studies have explored ceRNA regulatory network related to ferroptosis.

The ceRNA regulatory networks play important roles in the initiation and progression of cancer^34^. Here we identified a key ferroptosis-related ceRNA regulatory network comprising 4 lncRNAs, 6 miRNAs, and 4 ferroptosis-related genes in gastric cancer. This study found that *PWAR6*, *LINC00641*, *SNHG14*, and *PART1* are markedly downregulated and involved in ferroptosis of gastric cancer. Similarly, Yang et al. indicated that *LINC00641* is underexpressed in glioma cells, its overexpression inhibits cell proliferation but promoted apoptosis, and functions as a ceRNA in glioma cells by absorbing miR-4262 to regulate *NRGN*^35^. Moreover, we found that these four lncRNAs are regulated by six key miRNAs, such as miR-106b-5p, miR175-p, and miR-200b-3p, which are involved in the regulation of ferroptosis and possibly serve as candidate biomarkers for the prognosis and treatment of gastric cancer. Although all of these results provided evidence for the roles of these four key lncRNAs and six miRNAs in ferroptosis, more experimental studies are needed to confirm their mechanisms in gastric cancer.

## Conclusions

In conclusion, we analyzed the relationship between ncRNAs (lncRNAs and miRNAs) and ferroptosis in gastric cancer from the perspective of bioinformatics, and found an important ferroptosis-related ceRNA regulatory network, key lncRNAs and miRNAs that play critical roles in CG progression and affect the prognosis of GC patients. Hence, it will be important to validate the molecular mechanisms of these key lncRNA and miRNA regulators in ferroptosis of GC by experimental methods in the future. Our findings will provide references for proposing new biomarkers/targets of cancer therapy based on ferroptosis.

## Methods

### Data collection

RNA sequencing (RNAseq) data, miRNA sequencing (miRNAseq) data and corresponding clinical data of gastric cancer were collected from The Cancer Genome Atlas (TCGA) database (https://gdcportal.nci.nih.gov/). In this study, we downloaded the RNAseq data (raw counts and FPKM) of gastric adenomas and adenocarcinomas that contained the 343 tumor and 30 normal samples. FPKM is fragments per kilobase of exon model per million mapped fragments, reflects normalized gene expression level, and was transformed by log2. miRNA sequencing data of 410 tumor and 42 normal samples were obtained, which included the count data and normalized RPM (reads per million miRNA mapped reads) data. RPM values represent miRNA expression levels. In addition, GSE79973 and GSE78091 independent GC datasets were obtained from the GEO database (https://www.ncbi.nlm.nih.gov/geo/) and used as validation sets to validate the results of TCGA dataset analysis. GSE79973 dataset was generated using the GPL570 platform (Affymetrix Human Genome U133 Plus 2.0 Array) and normalized by the MAS 5.0 algorithm in GeneSpring Software 11.0 (Agilent Technologies, Santa Clara, CA, US). To further verify the reliability of our results, GEPIA, a web server for analyzing the gene expression profiling of 9,736 tumor and 8,587 normal samples from TCGA and GTEx projects^36^, was used to verify the expression of lncRNA and gene.

### Differential expression analysis of ferroptosis-related genes

FerrDb is a manually curated database for experimentally validated regulators and markers of ferroptosis and ferroptosisdisease associations^37^. The 259 ferroptosis-related genes were obtained from FerrDb database. To identify ferroptosis-related genes that are differentially expressed between tumor and normal tissues from GC patients, R package “DESeq2” was used^38^. The raw count of RNAseq data was used as input data in the DESeq2 package. Adjusted *P* values (padj) by false discovery rate (FDR) and log2 Fold Change (log2FoldChang) were used as screening parameters for differentially expressed genes.

### Prediction of ncRNAs interacting with ferroptosis-related genes

In order to obtain the upstream lncRNAs and miRNAs interacting with ferroptosis-related genes, miRNet, TargetScan and miRBase databases were used in this study^39–41^. These databases are widely applied in ncRNA studies, and their results have high confidence.

### Identification of key ncRNAs and construction of hub ceRNA regulatory network

The regulatory network between ncRNAs and ferroptosis-related genes were constructed by Cytoscape software platform^42^. The CytoHubba plugin in Cytoscape was utilized to identify hub ferroptosis-related genes in the network and construct hub subnetwork. The degrees of ferroptosis-related genes/ncRNAs in the network were calculated by topological methods, such as Degree, MCC, MNC, and clustering coefficients. The higher the degree, the more important the genes/ncRNAs.

### Kaplan-Meier survival analysis

Kaplan-Meier (KM) survival analysis was conducted to analyze the effect of ferroptosis-related gene expression on the survival of patients with GC. The gastric cancer samples were divided into high expression and low expression groups according to the median value of ferroptosis-related gene expression across all tumor samples. The log-rank test was applied to evaluate the difference in overall survival between the two groups of patients.

### Statistical analysis

Most of analyses were performed using R software for statistical computing and graphics. Based on Pearson's correlation coefficient, the correlation between expression level of miRNA and mRNA in GC was calculated using the miRNAseq and RNAseq data of GC in TCGA database. Similarly, the correlation between expression level of lncRNA and mRNA/miRNA was calculated. The expression levels of mRNA and lncRNA are log2-transformed FPKM values. The expression level of miRNA is log2-transformed RPM value. The *p* < 0.05 was considered statistically significant.

## Supplementary Information

Supplementary Information 1.

Supplementary Information 2.

Supplementary Information 3.

Supplementary Information 4.

## Data Availability

The datasets used and/or analyzed during the current study available from the corresponding author on reasonable request.

## Abbreviations

GC: Gastric cancer
ncRNA: Nonconding RNA
lncRNA: Long noncoding RNA
miRNA: MicroRNA
ceRNA: Competitive endogenous RNA
MRE: MiRNA response element
DElncRNA: Differentially expressed lncRNA
DEmiRNA: Differentially expressed miRNA
DEG: Differentially expressed gene
RPM: Reads per million miRNA mapped reads
KM analysis: Kaplan-Meier analysis
