# Supplementary material for: Identification of a key ceRNA network associated with ferroptosis in gastric cancer
Source: Sci Rep. 2022 Nov 22;12:20088. doi: 10.1038/s41598-022-24402-3 (PMC9684404; doi:10.1038/s41598-022-24402-3)
Supplement: Supplementary file 1 — Supplementary Information 1. [file 41598_2022_24402_MOESM1_ESM.pdf]

Supplementary Material

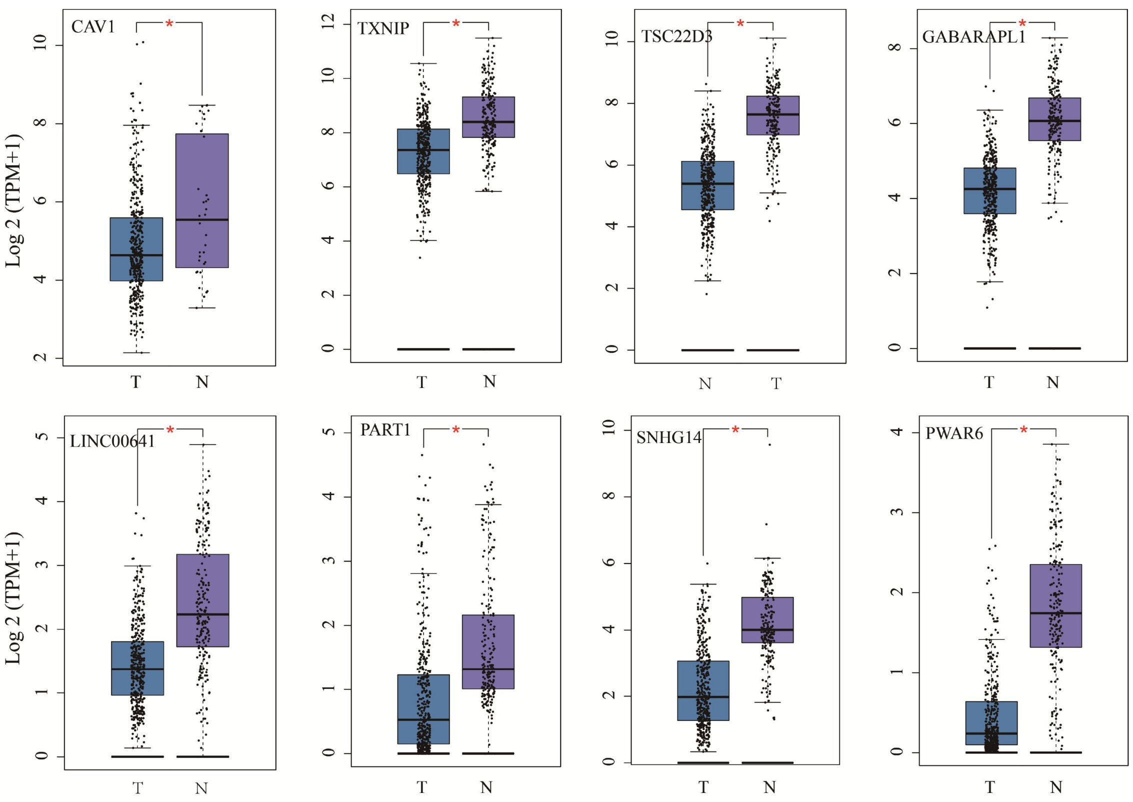

Fig.S1 The expression of four key ferroptosis-related genes and four key DElncRNAs in gastric cancer tissues and normal tissues based on GEPIA database. T: tumor tissues; N: normal tissues.

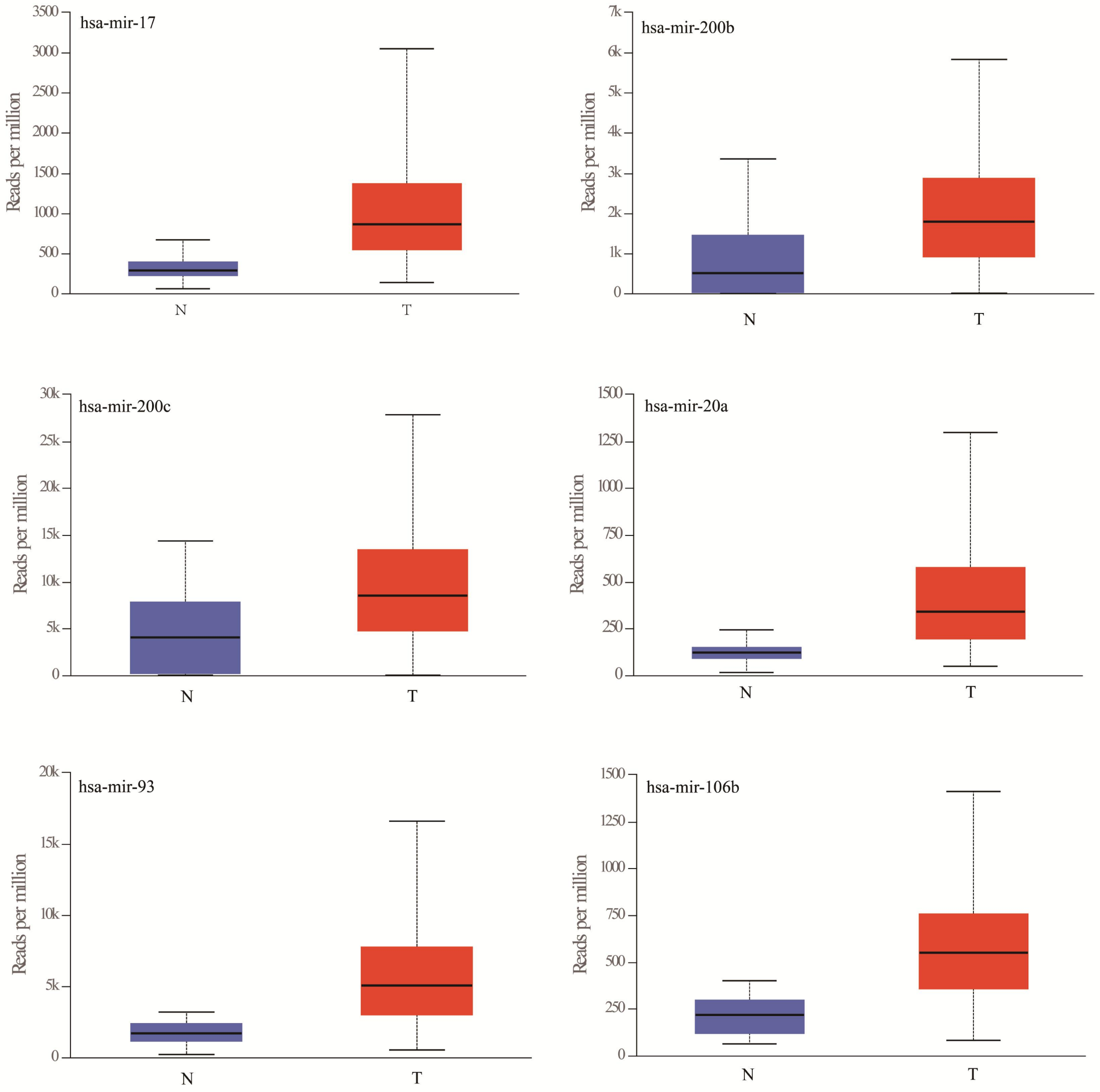

Fig.S2 The expression of six pivotal ferroptosis-related DEmiRNAs in gastric cancer tissues and normal tissues based on UALCAN database. T: tumor tissues; N: normal tissues.

Table S1 Top 20 enriched biological processes and pathways for 61 differentially expressed ferroptosis-related genes.

Term	Log10(*p*)	Gene Symbols
WP4313	-16.00	ALOX15,AKR1C1,AKR1C2,DPP4,HSPB1,SLC1A5,TFRC,AKR1C3,NOX1,NOX4,CA9,CAV1,PCK2,PRKAA2,TXNIP,ALB,NFE2L2,IFNG,IL6,DUOX1,CDO1,RGS4,HAMP,CDKN1A,MT3,GDF15,JUN,TP63
R-HSA-8953897	-12.64	ALB,ATF3,ATP6V1G2,CA9,CDKN1A,CDKN2A,HBA1,IL6,JUN,MT1G,MT3,NFE2L2,MAPK3,TXNIP,NOX4,TRIB3,CHAC1
GO:0031667	-12.64	ALB,ATF3,CDKN1A,NFE2L2,PCK2,PRKAA2,MAPK3,ZEB1,TFRC,ZFP36,AKR1C3,GDF15,GABARAPL1,HAMP
GO:0010035	-11.73	ALOX15,CAV1,HBA1,IL6,JUN,MT1G,MT3,NFE2L2,PRKAA2,MAPK3,TFRC,AKR1C3,TXNIP,HAMP,FANCD2,ANGPTL7,DUOX1,NOX1
GO:1901615	-11.06	ALOX12,ALOX15,ALOX15B,AKR1C1,AKR1C2,MT3,PCK2,PRKAA2,VLDLR,AKR1C3,PSAT1,DUOX1,MIOX,CDO1
M166	-10.52	ATF3,DUSP1,IFNG,IL6,JUN,MAPK3,JDP2,CDKN2A,MYB,CDKN1A,NFE2L2,CAV1,PRKAA2,NOX1,PCK2,HSPB1,STMN1,ALOX12,DPP4,FANCD2,ZEB1,TFRC,HAMP,ALB,ALOX15B,HBA1,DUOX1,ALOX15,ZFP36,NOX4,RRM2,DRD5,TXNIP,GABARAPL1,ATP6V1G2,MT1G,PSAT1,RGS4,HELLS,TSC22D3,SLC2A12,TP63,GDF15,ANGPTL7
GO:0010942	-9.70	ATF3,CAV1,CDKN1A,CDKN2A,HBA1,IFNG,IL6,JUN,MT3,TP63,AKR1C3,TXNIP,NOX1
GO:0019725	-9.56	CAV1,DRD5,IL6,MT1G,MT3,NFE2L2,PCK2,PRKAA2,MAPK3,TFRC,NOX1,NOX4,HAMP,IFNG
GO:0006631	-9.00	ALOX12,ALOX15,ALOX15B,AKR1C1,AKR1C2,PCK2,PRKAA2,AKR1C3,ALOXE3,ACSF2,ATF3,CDO1,PSAT1,ALB,CAV1,TRIB3,CDKN1A,DUSP1,MAPK3
GO:0072593	-8.94	ALOX12,DRD5,HBA1,MT3,NOX1,NOX4,DUOX1
GO:0071345	-8.85	ALOX15,DUSP1,HELLS,IFNG,IL6,MT3,NFE2L2,PCK2,MAPK3,TFRC,ZFP36,DUOX1,HAMP,CAV1,CDO1,AKR1C1,AKR1C2,PRKAA2,AKR1C3,TXNIP,TRIB3,FANCD2,DRD5
GO:0048871	-8.54	ALB,ALOX12,HSPB1,IL6,STMN1,PRKAA2,TP63,NOX4,HAMP,ALOXE3,CAV1,CDO1,NFE2L2,AKR1C3,TXNIP
GO:0048732	-8.11	ALOX15B,CAV1,CDO1,IL6,JUN,PCK2,MAPK3,AURKA,TP63,HAMP,CDKN1A,TFRC,NFE2L2,AKR1C3
M145	-7.92	ATF3,CAV1,CDKN1A,DUSP1,JUN,TP63,GDF15,IL6,MAPK3,RRM2,NFE2L2,NOX4,IFNG,STMN1,AURKA,TRIB3,CDKN2A,TFRC,ZFP36,ZEB1,HSPB1,MYB,AKR1C3,PRKAA2,ALOX15,PCK2,TXNIP,NOX1,HAMP
GO:0045596	-7.88	CAV1,IFNG,IL6,MT3,MYB,NFE2L2,RGS4,ZEB1,ZFP36,TP63,TRIB3,JDP2,ALOX15B,ATF3,FANCD2
hsa05208	-7.79	AKR1C1,AKR1C2,JUN,NFE2L2,MAPK3,AKR1C3,NOX1,NOX4,ALOX15,ATF3,CAV1,DUSP1,IL6,MT3,GDF15,VLDLR,ZFP36,DPP4,HSPB1,STMN1
GO:0031331	-7.65	CAV1,IFNG,IL6,NFE2L2,PRKAA2,MAPK3,AURKA,ZFP36,TRIB3,HAMP,ALOX12,CDKN2A,MT3,TP63,CHAC1,DUOX1,STMN1,DUSP1,NOX4,AKR1C3,CDO1,PCK2,HSPB1
GO:0051348	-7.13	CAV1,CDKN1A,CDKN2A,DUSP1,HSPB1,IFNG,ZFP36,TRIB3,JUN,DRD5,MT3,TXNIP,VLDLR,NOX4,PRKAA2,ALOX15B,IL6,ZEB1,TP63,CHAC1
GO:0006954	-7.04	ALOX15,CDO1,IFNG,IL6,JUN,NFE2L2,TFRC,NOX1,NOX4,HAMP,STMN1
WP3888	-6.62	ALB,CAV1,HSPB1,JUN,PRKAA2,MAPK3,TXNIP,NOX4,CHAC1,IL6

Log10(*p*): the p-value in log base 10.

Table S2 miRNAs interacted with ferroptosis-related genes and were negatively correlated with these genes.

Hub ferroptosis-related genes	Hub miRNAs	Pearson correlation
CAV1	miR-106b-5p, miR-17-5p, miR-200b-3p, miR-7-5p	Corr < -0.5; $p < 0.05$
TXNIP	miR-106b-5p, miR-17-5p, miR-200c-3p, miR-20a-5p, miR-93-5p	Corr < -0.5; $p < 0.05$
GABARAPL1	miR-17-5p	Corr < -0.5; $p < 0.05$
TSC22D3	miR-17-5p, miR-20a-5p, miR-335-5p	Corr < -0.5; $p < 0.05$

Table S3 lncRNAs interacted with miRNAs and were negatively correlated with these miRNAs.

Hub lncRNAs	Hub miRNAs	Pearson correlation
PART1	miR-17-5p, miR-106b-5p, miR-93-5p, miR-20a-5p	Corr < -0.39; $p < 0.05$
PWAR6	miR-17-5p, miR-106b-5p, miR-93-5p, miR-20a-5p	Corr < -0.31; $p < 0.05$
SNHG14	miR-106b-5p, miR-17-5p, miR-93-5p, miR-20a-5p	Corr < -0.33; $p < 0.05$
LINC00641	miR-200c-3p, miR-200b-3p	Corr < -0.40; $p < 0.05$
